# Oral Health and Fibromyalgia Syndrome: A Systemic Review

**DOI:** 10.3390/jfmk5010007

**Published:** 2020-01-25

**Authors:** Rosa De Stefano, Antonio Bruno, Maria Rosaria Anna Muscatello, Clemente Cedro, Alessandra Cicciù, Rosario Rullo, Michele Gaeta, Luca Fiorillo

**Affiliations:** 1 Department of Biomedical and Dental Sciences, Morphological and Functional Images, University of Messina, 98100 Messina ME, Italy; rsdestefano@libero.it (R.D.S.); Antonio.bruno@unime.it (A.B.); maria.muscatello@unime.it (M.R.A.M.); clemente.cedro@unime.it (C.C.); mgaeta@unime.it (M.G.); 2 Department of Biomedical and Dental Sciences, Morphological and Functional Images, School of Dentistry, University of Messina, 98100 Messina ME, Italy; alecicciu90@gmail.com; 3 Department of Biomedical and Surgical and Biomedical Sciences Naples University, 80100 Naples, Italy; rosario.rullo@unicampania.it

**Keywords:** fibromyalgia, temporomandibular joint, fatigue syndrome, chronic, oral health

## Abstract

Fibromyalgia is a syndrome currently considered idiopathic and multifactorial rheumatic that causes an increase in muscle tension and is characterized by muscle pain and chronic fibrous tissues—widespread, fluctuating and migrating—associated with stiffness, asthenia, cognitive disorders, insomnia or sleep disorders, alterations in sensitivity to stimuli. In affected patients, there may be anxiety or depressive disorder development. The aim of this study is, with the help of an interdisciplinary team, to evaluate the correlations between this syndrome and oral health. A literature review was conducted, analyzing the most common scientific databases, more than 200 studies were obtained. Subsequently to the application of filters and revision by the authors, only 18 articles were considered eligible for this review. From the results, it is clear that the correlations between fibromyalgia and oral health mainly concern pain in the oro-maxillofacial district, especially in the temporomandibular joint. This certainly could help for faster diagnosis of the syndrome, which is currently difficult to identify.

## 1. Introduction

### 1.1. Background

In the past, since 1800, fibromyalgia (FM) was already known but with many other names: in 1904, for example, the disease was called Fibrositis by William Richard Gowers. It occurs mainly in females in adulthood, although fibromyalgia is not uncommon in the pediatric age or during adolescence [1]. The prevalence of FM varies from 5 to 12%, depending on the population sampled. Females/males ratio is about 3:1 in studies that do not use tender points as a criterion. Major ethnic variations in prevalence have not been well documented. The prevalence of FM increases with age, rising in middle age (50–59 years) and then dropping off in the oldest age groups (80+ years). The average age of onset is between 30 and 50 years. The general population prevalence of FM in children and adolescents varies from 1.0% up to 6.2% [2]. Prevalence rates for FM in various conditions as temporomandibular (TM) disorders or TM pain and interstitial cystitis/painful bladder syndrome, endometriosis, chronic tension-type headache, chronic migraine headache, and chronic low back pain range from 20% to 65% [3]. Although not well known, it is indicated by recent statistics in second or third place among rheumatic diseases [4]. Its diagnosis and clinical features have long been controversial and its etiopathogenesis is still unknown [5,6,7,8]. This is not a mental disorder, although psychophysical stress and anxiety could affect it, and some specialists still see it as a diverse set of symptoms often treated as psychological, as they are partially similar to the physical effects of depressive disorder. In terms of history, important landmarks are the 1990 ACR (American College of Rheumatology) recognition of the condition as a chronic widespread pain condition involving trigger points (which set the stage for the next 20 years), the 2010/11 (then 2016) revisions to diagnosis which abandoned the trigger points and focused on the widespread pain and accompanying symptomology (and acknowledged comorbidities are common). ACR (American College of Rheumatology) classification carried out by Wolf et al. [2] in 1990, defined some criteria and symptoms about fibromyalgia syndrome. The symptoms in primary or secondary fibromyalgia were variable and different. Patients reported diffuse pain in different locations and different intensity. Among the characteristic symptoms were fatigue, difficulty in sleeping, morning stiffness. Some also reported paresthesia, headaches, and anxiety. There were also distinct modulating factors such as the quality of sleep or environmental humidity, which could influence musculoskeletal symptoms. The criteria for fibromyalgia were different and mainly related to the presence of tender points, the presence of 11 of 18 tender points was an accurate criterion for the primary or secondary-concomitant or combined diagnosis of fibromyalgia syndrome [2]. Today, a fibromyalgia syndrome diagnosis could be done according to AAPT ((ACTTION-APS) Addiction Clinical Trial Translations Innovations Opportunities and Networks–American Pain Society–Pain Taxonomy Criteria) [9]. This method provides an evidence-based diagnostic system for FM, but despite everything, there is not a specific test to diagnose this condition. The goal of the fibromyalgia Working Group was to apply the multidimensional diagnostic framework adopted by AAPT to FM and evaluate new approaches to the diagnosis of FM that might improve the recognition of FM in clinical practice. Briefly, in the AAPT taxonomy, there are five dimensions: dimension 1: core diagnostic criteria (Table 1); dimension 2: common features; dimension 3: common medical co-morbidities; dimension 4: neurobiological, psychosocial, and functional consequences; and dimension 5: putative neurobiological and psychosocial mechanisms, risk factors, and protective factors.

In dimension 3, they defined that FM is associated with many comorbidities that may be categorized as other somatic pain disorders, psychiatric conditions, sleep disorders, rheumatic diseases, and other conditions. It is commonly conjectured that many of these associations are a central sensitization result, but this mechanism could not explain all associations. Chronic fatigue syndrome is a condition that has considerable overlap with FM, with the predominance of pain an identifier of FM. Among the somatic pain conditions that associate with FM, some recognized are irritable bowel syndrome, chronic pelvic pain, and interstitial cystitis, chronic head and orofacial conditions such as temporomandibular disorder, otologic symptoms, chronic headaches, and migraine disorder [9,10].

Fibromyalgia frequently presents pain on the maxillary or mandibular district and in these cases, the symptomatology is confused with arthrosis or dysfunction of the temporomandibular joint. Central Sensitivity System (CSS) seems to be a useful paradigm and appropriate terminology for FMS and related conditions [11].

### 1.2. Objectives

The purpose of this review is to highlight all the possible correlations between fibromyalgia and oral health. An analysis of the literature was conducted to bring to light all these tests. Being able to identify pathology, difficult to diagnose as fibromyalgia, even starting from alterations in oral health, could be an advantage for clinicians.

## 2. Materials and Methods 

### 2.1. Protocol and Registration

**Figure 1:**
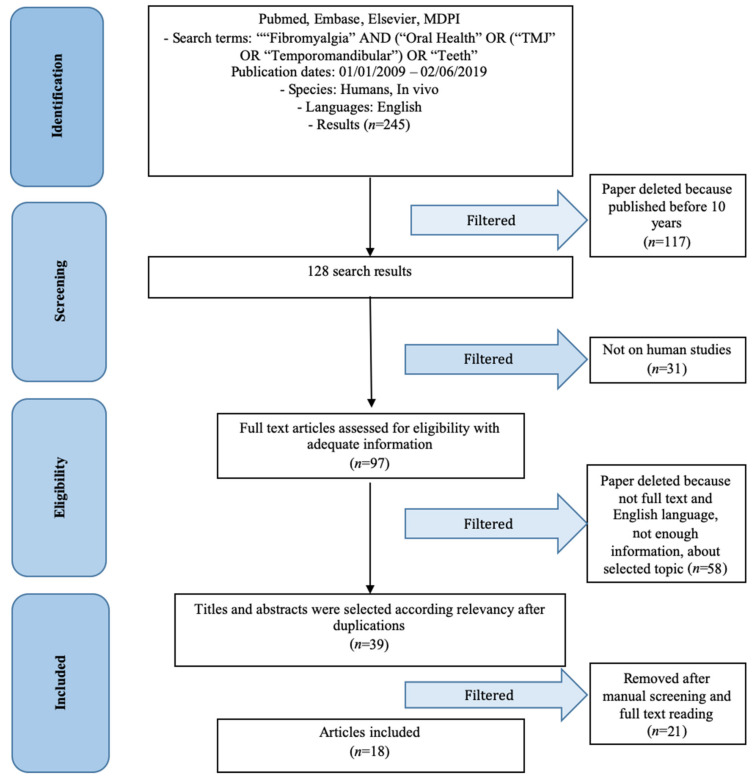
PRISMA (transparent reporting of systematic reviews and meta-analyses) Flow Diagram. The flow diagram depicts the flow of information through the different phases of a systematic review. It maps out the number of records identified, included and excluded, and the reasons for exclusions.

This systematic review of the literature was conducted based on the PRISMA (Transparent Reporting of Systematic Reviews and Meta-Analyses) statement. The PRISMA checklist for the conduct of the review, analysis, and editing of the manuscript was followed (Figure 1). Furthermore, the main question is in accordance with the PICO (Population/Intervention/Comparison/Outcome) study design. The systematic review was recorded in the international PROSPERO (International Prospective Register of Systematic Reviews) database with the following number 140,739 on 30 June 2019.

### 2.2. Eligibility Criteria

The obtained results from the research were filtered based on eligibility and exclusion criteria as follows:

Eligibility criteria:

- Study conducted on humans
- Studies containing information on fibromyalgia
- Studies containing information on oral health

Exclusion criteria:

- Studies older than 10 years
- Animal studies
- In vitro studies
- Studies on patients suffering from other pathologies (besides fibromyalgia and oral alterations)
- Studies not accessible (title or abstract)

### 2.3. Information Sources

The sources of information for data collection are represented by search engines in the medical/scientific field Pubmed, Embase, Elsevier, MDPI. In addition, a manual literature search was conducted on textbooks, without obtaining recent information (Figure 1).

### 2.4. Search

The search was conducted on search engines, according to the previous paragraph. It was conducted several times, making sure not to get errors due to software processing. The keywords used are the following: “fibromyalgia” AND “oral health” OR “TMJ” OR “temporomandibular” OR “teeth”. Search keywords have been chosen so as to limit the number of errors and increase the number of results. The main question is:

- Have Fibromyalgia Syndrome-affected patients an increased risk for oral health?

### 2.5. Study Selection

The studies, once obtained with electronic search engine filters, were manually reviewed independently by two authors (L.F. and R.R.) to obtain the necessary information. 

### 2.6. Data Collection Process

The authors conducted an independent review of the results, and after reading the titles and abstracts, they evaluated their eligibility to be included in the systematic review.

### 2.7. Data Items 

The review was conducted in accordance with the PRISMA statement, and the main question was conducted in accordance with the PICO guidelines.

### 2.8. Risk of Bias in Individual Studies 

The risk of bias has been evaluated thanks to ROBIS guidelines (Risk of Bias in Systematic Review) [12], according to the indications of Higgins et al. [13,14,15]. Data collection was carried out in order to limit the risk of bias. Furthermore, a table with the risk of individual bias of the results has been provided (Table 2).

### 2.9. Summary Measures 

The main parameters taken into consideration concern oral health abnormalities related to fibromyalgia. In particular, as shown in the table in the next section and in the “study characteristics” section, the characteristics considered are:

- Authors and Year—author and year of the study (in order of year, from the most recent)
- Fibromyalgia—diagnosis confirmed or not
- Oral Health:
  ○ Bone tissue—bone anomalies
  ○ Soft Tissue—anomalies on gums, mucous membranes, tongue, and muscles
  ○ TMJ—temporomandibular joint anomalies
- Statistic—statistical data on the findings
- Sample and Methods—sample size and used methods or tests

Results: notes on the results of the study (Table 2, Table 3 and Table 4)

### 2.10. Synthesis of Results

According to PRISMA statement, a synthesis of results table has been provided. In this table (Table 2) Information about sample and methods are showed, there is also a column showing additional data regarding the topics covered by the manuscript examined (✔).

## 3. Results

### 3.1. Study Selection

Research on the different scientific electronic engines provided 245 results, no results were obtained from the search on textbooks in the field of oral medicine and fibromyalgia. After the application of a first search parameter (studies in the last 10 years), only 128 studies were considered. There were 97 human studies, of these after a full-text manual screening, only 18 studies were considered. These 18 papers were included in the systematic review (Table 3 and Table 4).

### 3.2. Study Characteristics 

For each study, all oral health alterations in fibromyalgia patients were considered. These are expressed in the form of results in the next paragraph. The characteristics considered concerns the different fields of oral health.

### 3.3. Risk of bias Within Studies 

According to PRISMA statement a risk of bias analysis has been conducted (Table 3).

### 3.4. Results of Individual Studies and Synthesis of Results 

Results of individual studies, has been provided in Table 4, according to PRISMA statement.

## 4. Discussion

### 4.1. Summary of Evidence

A manual review of the articles has produced interesting results. The results are discussed below with the aim of explaining the main data (Table 4) and above all promoting the knowledge and correlations between FM and TMD. Furthermore, it could be deduced that FM has correlations not only with TMJ but also with the cranial cervical and mandibular skull district [34]. According to Velly et al. [16], there is a correlation between fibromyalgia, depression and TMJ disorders. According to them, fibromyalgia pain should have a central role and could be considered when evaluating a treatment for TMJ disorders [35]. Hoffmann et al. [17] evaluated comorbidities associated with TMJD. Fibromyalgia is one of these, and there are significant data about its correlation with TMJD. In a Karibe et al. [18] study, TMJ disfunction, myofascial pain, neuropathic pain and fibromyalgia were compared as pain intensity and difficulty in performing activities of daily living (ADL). According to Kindler et al. [19], some patients with central nervous system (CNS) alteration, show TMJD; fibromyalgia patients have this condition too. Alonso-Bianco et al. [20] compared differences in the prevalence and anatomical localization of active TrPs (tender points) in women with fibromyalgia or myofascial TMJD. For example, according to the authors, there are significant differences on the localization of TrPs between these pathologies: temporalis and masseter muscles are more active in TMJD than in fibromyalgia. These differences could help clinicians during an early diagnosis. Suma et al. [21] in their 2012 literature review showed how myofascial pain syndrome, chronic fatigue syndrome, and fibromyalgia “causes” [21] TMD and TMJ pain. TMJ pain could be caused by stress, infection, parafunctional habits, inflammation, or other factors like psychosocial conditions [36,37,38,39,40]. According to De Rossi et al. [22], fibromyalgia causes diffuse masticatory muscle pain and disorders, and they could be resolved using fibromyalgia drugs. There are different drugs that could be beneficial to masticatory muscle pain: SSRI, NSAID, SNRI, TCAs, muscle relaxants (Table 5). According to de Siqueira et al. [23], fibromyalgia affected patients show sensorial abnormalities, TMJ abnormalities, and pain. Cassisi et al. [24] proposed a flow chart for fibromyalgia, myofascial pain, chronic fatigue syndrome diagnosis. According to the authors, the starting point is represented by TMJ pain, TMJ only or TMJ and another. Subsequently, it is necessary to evaluate trauma, parafunctions, infections, trigger points or psychological aspects. Jin et al. [25] in their study proposed orofacial management of fibromyalgia patients. It includes accurate analysis of masticatory muscles and TMJ before proceeding with pharmacological therapies. According to Dahan et al. [26], TMD patients with FM experience more intense and prolonged pain than TMD patients without FM. According to Eisenlohr-Moul et al. [27], TMD and FM patients showed greater parasympathetic decline suggesting an autonomic stance that is supporting defensive than engagement. Furquim et al. [28] aimed at showing physio-pathologic mechanisms associated with TMD. TMD symptoms are managed by complex mechanisms influenced by the autonomic systems and depend on an individual’s adaptation. Gui et al. [29] showed how TMD and FM syndrome are not even coexisting but could have similar predisposing triggering factors. Cummiford et al. [30] evaluated the use of tDCS on FM patients as it could produce analgesia. This transcranial stimulation may produce analgesia on TMD too. Fujarra et al. [31] showed oro-facial symptoms of FM patients, evaluating joint and muscle disorders with associated pain. Robinson et al. [32] evaluated differences between TMD and CFS, CFS is a syndrome associated with hypersensitivity of the CNS, and it is a similar condition to FM. In a sample of this study, 50% of CFS patients with TMD reported fibromyalgia too. There is confirmed comorbidity between oro-facial pain and CFS. Losert-Bruggner et al. [33] evaluated the correlation between FM and craniocervical disorders and craniomandibular disorders. They evaluated pain through a pain index after a bite splint therapy (myocentric) and a neuromuscular relaxation method. Any improving symptoms were registered in a significant manner and authors say that these patients could take advantage by an interdisciplinary approach. Fibromyalgia therefore has clear correlations at the level of TMJ and oral health. In particular, as can be seen from the results, patients with TMD and FM show an increase in symptoms [41,42,43,44,45]. Fibromyalgia is diagnosed due to the exclusion of other pathologies and subsequent palpation of the tender points, although it is the patient’s overall symptomatic picture that leads to diagnosis. Mainly affected by pain are: all the districts of the spine, the shoulders, the pelvic girdle, arms, wrists, thighs [36,46,47,48,49,50,51,52,53,54,55,56]. Chronic pain, which often occurs at intervals, is associated with different symptoms, especially cognitive disorders, generally identified with the term fibro fog, mood and sleep disorders, as well as fatigue or chronic fatigue. The non-response to common painkillers, as well as the “migrant” character of the pains, are peculiar to fibromyalgia. The main manifestation is a sensation of pain, fatigue and temporary disturbances of cognitive functions, difficulty falling asleep or sleeping. A recent study has shown the presence of an alteration at the anatomical level: an excessive innervation in the hands, which concerns the nerves involved in regulation (opening and closing), caused by local arteriovenous shunts. Alterations of numerous neurotransmitters have been demonstrated and confirmed, reflecting the origin of fibromyalgia in the central nervous system, a species of serotonin. All drugs that have been shown to be effective act on the central nervous system. One of the effects of neurotransmitter dysfunction, and in particular of serotonin and noradrenaline, is the hyperactivity of the neurovegetative nervous system. Discussed, given the involvement of the CNS and not only of the peripheral nervous system, is the possible association—in the case of fibromyalgia that is characterized by acute pain—with peripheral small-fiber neuropathy. Small-fiber neuropathy has unknown causes; when it is not related to manifest diseases (lupus erythematosus, sarcoidosis, HIV or Lyme disease [54,57]) it can be linked to celiac disease [55], allergies, non-celiac gluten sensitivity. Fibromyalgia is not a psychiatric illness and anxiety problems make it worse. The risk of developing anxiety disorders is about five times higher than for non fibromyalgics. 

**Figure 2:**
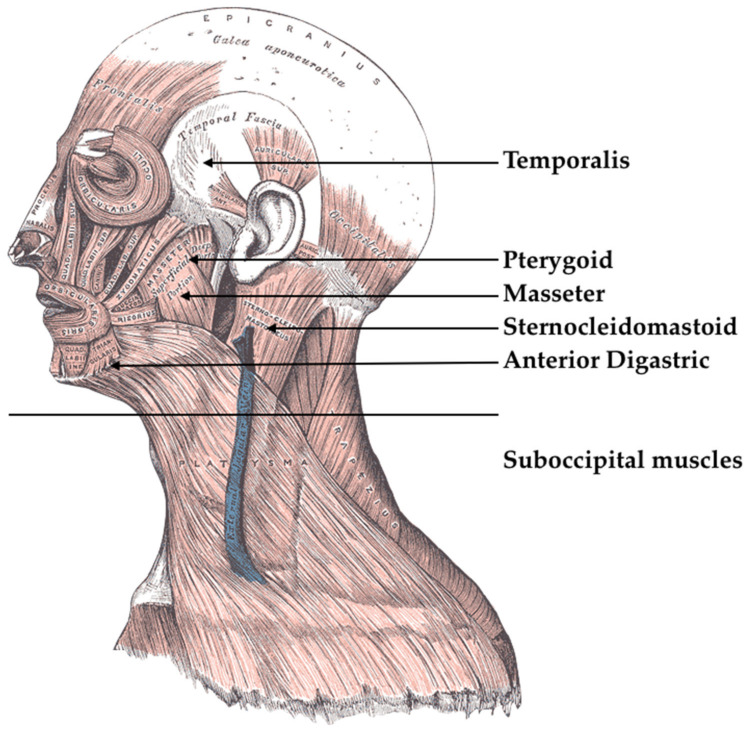
Fibromyalgia (FM) involved head-neck muscles. Henry Vandyke Carter (Public domain).

There are several syndromes and diseases which, due to their characteristics, can be similar to fibromyalgia as symptoms, and which will be excluded, even though they may be concomitant; among them, there are mainly:

Sjögren syndrome, due to the low nuclear antibodies contained, multiple sclerosis, other systemic neurological syndromes, arthritis and arthrosis [37,39,58,59,60,61,62,63]. Due to the impossibility of formulating a diagnosis based on medical evidence, and especially considering the equivocal nature of fibromyalgia, there is no universally adopted therapy whose efficacy is scientifically proven, different drugs are used [59], such as non-steroidal anti-inflammatory drugs (NSAIDs), selective serotonin reuptake inhibitors (SSRIs), cannabinoids, and muscle relaxants. This syndrome is not a fatal disease. The causes are multiple and, in the same individual, a single triggering factor. On the one hand, there are psycho-social factors, such as behavior towards diseases, cognitive, and emotional aspects; on the other hand, biological factors, such as predisposition and individual susceptibility, as for other syndromes or alterations as irritable bowel syndrome; alterations of the motility of the digestive tract, sensitivity of the viscera, subjective perception of pain, bacterial flora and intestinal infections [64,65,66,67]. According to Table 4 and Table 5, some useful clinical evidence could be highlighted. First of all, fibromyalgia patients centrally generated pain plays an important role in TMJ pain and TMJ disorders, this could be clinical information for differential diagnosis, therefore, there are different trigger points between TMD and FM. Neuropathy and CNS hypersensitivity could play a role in TMD. As for therapy, it could be pharmacological, and some fibromyalgia used drugs, at the same time, reduce the TMD symptoms (Figure 2).

### 4.2. Limitation

The limitations of this study may be related to the sources, only articles from the most important scientific databases were evaluated, and the impact factors of the journals were also revised, ensuring the quality of the source. However, one limitation of this study is that of not having enough data in order to produce a univocal statistic of the results.

## 5. Conclusions

The results show an important correlation between fibromyalgia and alterations affecting the craniomaxillofacial and craniomandibular district. This once again demonstrates an important correlation between the temporomandibular joint and the vertebral column with all the systemic implications arising from it. Some FM symptoms could be useful for early diagnosis. Different studies assess the presence of pain in the area of the TMJ, often associated with abuse of analgesic drugs by patients. Furthermore, occlusal and TMJ-sensitive abnormalities or muscle disorders are reported. Patients also appear to respond well to therapy with direct transcranial stimulation, certainly, an interdisciplinary approach is needed. Fibromyalgia is a widespread pathology, difficult to diagnose, to be able to easily understand oral symptoms, the TMJ could be the first step towards early diagnosis. An important future clinical study could focus on all oral manifestations, from dental pain to those associated with the mucous membranes and, therefore, to the muscles and bones of the maxillofacial district.

## Figures and Tables

**Table 1:** AAPT Dimension 1 diagnostic criteria.

Dimension 1 Criteria
Dimension 1: Core Diagnostic Criteria
MSP defined as 6 or more pain sites from a total of 9 possible sites
Moderate to severe sleep problems OR fatigue
MSP plus fatigue or sleep problems must have been present for at least 3 months

**Table 2:** Synthesis of results (see Section 2.9).

Authors and Year	Fibromyalgia	Oral Health			Statistic	Sample and Methods
		Bone Tissue	Soft Tissue	TMJ		
Velly et al., 2010 [16]	✔		✔	✔	Significant	485 patients–Graded Chronic Pain Scale (GCPS).
Hoffmann et al., 2011 [17]	✔			✔	*p* = 0.0001	1511 patients.
Karibe et al., 2011 [18]	✔			✔	*p* < 0.01	237 patients–Pain and Activity of Daily Living (ADL)
Kindler et al., 2011 [19]	✔			✔	Review	Review
Alonso-Bianco et al., 2012 [20]	✔		✔	✔	Significant	20 patients–Active trigger points (TrPs) localization
Suma et al., 2012 [21]	✔		✔	✔	Review	Review
De Rossi et al., 2013 [22]	✔		✔	✔	Review	Review
De Siqueira et al., 2013 [23]	✔	✔	✔	✔	Significant	116 patients–pain evaluation, orofacial characteristics, oral health, sensivity to muscular palpation.
Cassisi et al., 2014 [24]	✔		✔	✔	Review	Review
Jin et al., 2014 [25]	✔		✔	✔	Review	Review
Dahan et al., 2015 [26]	✔		✔	✔	Significant	224 patients–TMD comorbidities and which causes pain increase and duration
Eisenlohr-Moul et al., 2015 [27]	✔				Significant	43 patients–baseline assessment of respiratory sinus arrhythmia, parasympathetic assessment during a questionnaire period
Furquim et al., 2015 [28]	✔				Review	Review
Gui et al., 2015 [29]	✔				Review	Review
Cummiford et al., 2016 [30]	✔				Significant	12 patients-Pain-Transcranial direct current stimulation (tDCS) use
Fujarra et al., 2016 [31]	✔		✔	✔	Significant	53 patients–TMD diagnosis and Visual Analogue Scale (VAS)
Robinson et al., 2016 [32]	✔		✔	✔	Review	Review
Losert-Bruggner et al., 2018 [33]	✔		✔	✔	Significant	555 patients–Pain index–bite splint therapy and neuromuscular relaxation measures

**Table 3:** Risk of bias results table.

Authors and Year	Risk of Bias			
	Low	Moderate	High	Unclear
Velly et al., 2010 [16]	✔			
Hoffmann et al., 2011 [17]	✔			
Karibe et al., 2011 [18]		✔		
Kindler et al., 2011 [19]				✔
Alonso-Bianco et al., 2012 [20]		✔		
Suma et al., 2012 [21]	✔			
De Rossi et al., 2013 [22]	✔			
De Siqueira et al., 2013 [23]		✔		
Cassisi et al., 2014 [24]	✔			
Jin et al., 2014 [25]	✔			
Dahan et al., 2015 [26]		✔		
Eisenlohr-Moul et al., 2015 [27]			✔	
Furquim et al., 2015 [28]	✔			
Gui et al., 2015 [29]	✔			
Cummiford et al., 2016 [30]			✔	
Fujarra et al., 2016 [31]		✔		
Robinson et al., 2016 [32]				✔
Losert-Bruggner et al., 2018 [33]	✔			

**Table 4:** Synthesis of results according to Section 2.9.

Authors and Year	Results
Velly et al., 2010 [16]	According to the authors, the risk associated with baseline fibromyalgia was moderate. Centrally generated pain plays a role in TMJ disorders (TMJD) and muscle pain.
Hoffmann et al., 2011 [17]	According to the authors, TMJD-affected individuals were on average 41 years old and predominantly female (90%). Patients reported moderate-to-severe intensity of pain. In this case-control comparison study, a higher frequency of depression, fatigue and fibromyalgia is correlated to TMJD. Fibromyalgia was a comorbid condition
Karibe et al., 2011 [18]	Fibromyalgia patients and Neuropathy patients significantly reported greater pain intensity in the TMJ area.
Kindler et al., 2011 [19]	Central nervous system alteration could reflect TMJD
Alonso-Bianco et al., 2012 [20]	TrPs were identified by palpation, and patients referred to pain in these areas. The number of active TrPs was significantly higher in TMD patients than in FMS ones. Women with FMS referred to pain in these areas more than those with TMD. Significant differences within the center of gravity coordinates of TrPs were found in TMD and FMS. There are different TrPs localization between myofascial temporomandibular disorders (TMD) and fibromyalgia patients.
Suma et al., 2012 [21]	According to the authors, in fibromyalgia patients, TMD and pain remain a recurring problem and they need a correct diagnosis and management to resolve this problem. Often therapies are dictated by the cause as inflammation, trauma, aging or parafunctional habits.
De Rossi et al., 2013 [22]	According to the authors, many medicaments used for fibromyalgia could be used for TMJD.
De Siqueira et al., 2013 [23]	Sensorial anomalies were observed in neuropathic or somatic pain patients, as in fibromyalgia patients. According to the authors, the majority of patients had pain upon craniofacial muscle palpation. Persistent idiopathic facial pain and temporomandibular disorders were associated with a low threshold for pain perception. (*p* < 0.002)
Cassisi et al., 2014 [24]	Pain in fibromyalgia or TMJD is caused by CNS hypersensitivity. Pharmacological and non-pharmacological therapies have been suggested for the treatment of these conditions.
Jin et al., 2014 [25]	Masticatory muscle pain and TMJ pain could be associated with fibromyalgia, despite the internal derangements of the TMJ.
Dahan et al., 2015 [26]	There was a positive association between the number of comorbidities present and TMD pain duration (*p* < 0.01), also the presence of migraine was positively associated. TMD and fibromyalgia are associated with an increase of TMJ pain intensity and duration.
Eisenlohr-Moul et al., 2015 [27]	TMD and fibromyalgia showed a higher parasympathetic decline during a psychosocial assessment.
Furquim et al., 2015 [28]	TMD is managed by complex mechanisms by the autonomic nervous systems as is fibromyalgia.
Gui et al., 2015 [29]	TMD and fibromyalgia (FM) are not merely coexisting conditions, but they have a series of similar characteristics and predisposing triggering factors.
Cummiford et al., 2016 [30]	Transcranial direct current stimulation (tDCS) may produce analgesia by altering thalamic connectivity while there may be a placebo response. Stronger baseline functional connectivity between M1-VL (left primary motor cortex- ventral lateral) thalamus, S1-anterior insula (primary somatosensory cortices), and VL thalamus-PAG (periaqueductal) predicted greater analgesia after sham and real tDCS. Sham treatment (compared with baseline) reduced FC between the VPL (ventral posterolateral) thalamus, S1, and the amygdala. Real tDCS (compared with sham treatment) reduced FC between the VL thalamus, medial prefrontal, and supplementary motor cortices. Interestingly, decreased FC between the VL/VPL thalamus and posterior insula, M1, and S1 correlated with reductions in clinical pain after both sham and active treatments.
Fujarra et al., 2016 [31]	All patients showed TMD and muscle disorders, with limited opening and TMJ disc displacement. According to the authors, Myofascial pain could be associated with mouth opening limitation (*p* = 0.038); right disc displacement with reduction (*p* = 0.012) and jaw stiffness (*p* = 0.004) were predominant in the facial pain group. Myofascial pain without mouth opening limitation (*p* = 0.038) and numbness/burning were more common in the facial or generalized pain sample group.
Robinson et al., 2016 [32]	Chronic painful TMD is a central sensitivity syndrome related to the hypersensitivity of the CNS. Similar conditions are Chronic Fatigue Syndrome (CFS) and FM.
Losert-Bruggner et al., 2018 [33]	Patients with cranio-cervical disfunctions and craniomandibular disorders benefit from interdisciplinary treatment. Using myocentric bite splint therapy and therapy with oral orthosis in combination with neuromuscular relaxation measures, an improvement of physical symptoms was seen in 84% of CMD-FMS patients, and improvement of the symptoms in the jaw was achieved in 77% of cases.

**Table 5:** Medications used for fibromyalgia that may be beneficial for masticatory muscle pain according to De Rossi et al. [22].

Drug Category	Effect
Tricyclic antidepressants (TCAs)	Moderately helpful for pain, more side effects (xerostomia, fatigue)
Serotonin-selective reuptake inhibitors	Fewer side effects than TCAs, more effective for anxiety/depression than for pain
Muscle relaxants	Moderately helpful for local muscle pain, more side effects (xerostomia, sedation)
Serotonin-norepinephrine reuptake inhibitors	Moderately helpful for fibromyalgia-related pain, low-potency opioids, moderately helpful for fibromyalgia-related pain
NSAIDs	Helpful for acute inflammatory pain but not chronic muscle pain or fibromyalgia-related pain

## References

[1] Sumpton J.E., Moulin D.E. (2014). Fibromyalgia. Handb. Clin. Neurol..

[2] Wolfe F., Smythe H.A., Yunus M.B., Bennett R.M., Bombardier C., Goldenberg D.L., Tugwell P., Campbell S.M., Abeles M., Clark P. (1990). The American College of Rheumatology 1990 Criteria for the Classification of Fibromyalgia. Report of the Multicenter Criteria Committee. Arthritis Rheumatol..

[3] Fitzcharles M.-A., Perrot S., Häuser W. (2018). Comorbid fibromyalgia: A qualitative review of prevalence and importance. Eur. J. Pain.

[4] Hauser W., Ablin J., Fitzcharles M.A., Littlejohn G., Luciano J.V., Usui C., Walitt B. (2015). Fibromyalgia. Nat. Rev. Dis. Primers.

[5] Quintner J.L., Cohen M.L. (1999). Fibromyalgia falls foul of a fallacy. Lancet.

[6] Henriksson K.G., Bengtsson A., Larsson J., Lindstrom F., Thornell L.E. (1982). Muscle biopsy findings of possible diagnostic importance in primary fibromyalgia (fibrositis, myofascial syndrome). Lancet.

[7] Cohen M.L., Quintner J.L. (1993). Fibromyalgia syndrome, a problem of tautology. Lancet.

[8] Szychlinska M.A., Yamakado K., Castorina A., Ljubisavljevic M. (2017). The “Journal of Functional Morphology and Kinesiology” Journal Club Series: Highlights on Recent Papers in Musculoskeletal Disorders. J. Funct. Morphol. Kinesiol..

[9] Arnold L.M., Bennett R.M., Crofford L.J., Dean L.E., Clauw D.J., Goldenberg D.L., Fitzcharles M.A., Paiva E.S., Staud R., Sarzi-Puttini P. (2019). AAPT Diagnostic Criteria for Fibromyalgia. J. Pain.

[10] Staud R. (2006). Biology and therapy of fibromyalgia: Pain in fibromyalgia syndrome. Arthritis Res. Ther..

[11] Yunus M.B. (2008). Central sensitivity syndromes: A new paradigm and group nosology for fibromyalgia and overlapping conditions, and the related issue of disease versus illness. Semin. Arthritis Rheumatol..

[12] Whiting P., Savovic J., Higgins J.P.T., Caldwell D.M., Reeves B.C., Shea B., Davies P., Kleijnen J., Churchill R. (2018). ROBIS: A new tool to assess risk of bias in systematic reviews was developed. Recenti Prog. Med..

[13] Savovic J., Turner R.M., Mawdsley D., Jones H.E., Beynon R., Higgins J.P.T., Sterne J.A.C. (2018). Association Between Risk-of-Bias Assessments and Results of Randomized Trials in Cochrane Reviews: The ROBES Meta-Epidemiologic Study. Am. J. Epidemiol..

[14] Mansournia M.A., Higgins J.P., Sterne J.A., Hernan M.A. (2017). Biases in Randomized Trials: A Conversation Between Trialists and Epidemiologists. Epidemiology.

[15] Higgins J.P., Altman D.G., Gotzsche P.C., Juni P., Moher D., Oxman A.D., Savovic J., Schulz K.F., Weeks L., Sterne J.A. (2011). The Cochrane Collaboration’s tool for assessing risk of bias in randomised trials. BMJ.

[16] Velly A.M., Look J.O., Schiffman E., Lenton P.A., Kang W., Messner R.P., Holcroft C.A., Fricton J.R. (2010). The effect of fibromyalgia and widespread pain on the clinically significant temporomandibular muscle and joint pain disorders--a prospective 18-month cohort study. J. Pain.

[17] Hoffmann R.G., Kotchen J.M., Kotchen T.A., Cowley T., Dasgupta M., Cowley A.W. (2011). Temporomandibular disorders and associated clinical comorbidities. Clin. J. Pain.

[18] Karibe H., Goddard G., McNeill C., Shih S.T. (2011). Comparison of patients with orofacial pain of different diagnostic categories. Cranio.

[19] Kindler L.L., Bennett R.M., Jones K.D. (2011). Central sensitivity syndromes: Mounting pathophysiologic evidence to link fibromyalgia with other common chronic pain disorders. Pain Manag. Nurs..

[20] Alonso-Blanco C., Fernandez-de-Las-Penas C., de-la-Llave-Rincon A.I., Zarco-Moreno P., Galan-Del-Rio F., Svensson P. (2012). Characteristics of referred muscle pain to the head from active trigger points in women with myofascial temporomandibular pain and fibromyalgia syndrome. J. Headache Pain.

[21] Suma S., Veerendra Kumar B. (2012). Temporomandibular disorders and functional somatic syndromes: Deliberations for the dentist. Indian J. Dent. Res..

[22] De Rossi S.S., Stern I., Sollecito T.P. (2013). Disorders of the masticatory muscles. Dent. Clin. N. Am..

[23] De Siqueira S.R., Teixeira M.J., de Siqueira J.T. (2013). Orofacial pain and sensory characteristics of chronic patients compared with controls. Oral Surg. Oral Med. Oral Pathol. Oral Radiol..

[24] Cassisi G., Sarzi-Puttini P., Casale R., Cazzola M., Boccassini L., Atzeni F., Stisi S. (2014). Pain in fibromyalgia and related conditions. Reumatismo.

[25] Jin H., Patil P.M., Sharma A. (2014). Topical review: The enigma of fibromyalgia. J. Oral Facial Pain Headache.

[26] Dahan H., Shir Y., Velly A., Allison P. (2015). Specific and number of comorbidities are associated with increased levels of temporomandibular pain intensity and duration. J. Headache Pain.

[27] Eisenlohr-Moul T.A., Crofford L.J., Howard T.W., Yepes J.F., Carlson C.R., de Leeuw R. (2015). Parasympathetic reactivity in fibromyalgia and temporomandibular disorder: Associations with sleep problems, symptom severity, and functional impairment. J. Pain.

[28] Furquim B.D., Flamengui L.M., Conti P.C. (2015). TMD and chronic pain: A current view. Dent. Press J. Orthod..

[29] Gui M.S., Pimentel M.J., Rizzatti-Barbosa C.M. (2015). Temporomandibular disorders in fibromyalgia syndrome: A short-communication. Rev. Bras. Reumatol..

[30] Cummiford C.M., Nascimento T.D., Foerster B.R., Clauw D.J., Zubieta J.K., Harris R.E., DaSilva A.F. (2016). Changes in resting state functional connectivity after repetitive transcranial direct current stimulation applied to motor cortex in fibromyalgia patients. Arthritis Res..

[31] Fujarra F.J., Kaziyama H.H., Siqueira S.R., Yeng L.T., Camparis C.M., Teixeira M.J., Siqueira J.T. (2016). Temporomandibular disorders in fibromyalgia patients: Are there different pain onset?. Arq NeuroPsiquiatr.

[32] Robinson L.J., Durham J., Newton J.L. (2016). A systematic review of the comorbidity between Temporomandibular Disorders and Chronic Fatigue Syndrome. J. Oral Rehabil..

[33] Losert-Bruggner B., Hulse M., Hulse R. (2018). Fibromyalgia in patients with chronic CCD and CMD—A retrospective study of 555 patients. Cranio.

[34] Isaia B., Ravarotto M., Finotti P., Nogara M., Piran G., Gamberini J., Biz C., Masiero S., Frizziero A. (2019). Analysis of Dental Malocclusion and Neuromotor Control in Young Healthy Subjects through New Evaluation Tools. J. Funct. Morphol. Kinesiol..

[35] Bruno A., Mico U., Lorusso S., Cogliandro N., Pandolfo G., Caminiti M., Zoccali R.A., Muscatello M.R. (2013). Agomelatine in the treatment of fibromyalgia: A 12-week, open-label, uncontrolled preliminary study. J. Clin. Psychopharmacol..

[36] Fiorillo L., Cervino G., Herford A.S., Lauritano F., D’Amico C., Lo Giudice R., Laino L., Troiano G., Crimi S., Cicciu M. (2018). Interferon Crevicular Fluid Profile and Correlation with Periodontal Disease and Wound Healing: A Systemic Review of Recent Data. Int. J. Mol. Sci.

[37] Isola G., Ramaglia L., Cordasco G., Lucchese A., Fiorillo L., Matarese G. (2017). The effect of a functional appliance in the management of temporomandibular joint disorders in patients with juvenile idiopathic arthritis. Minerva Stomatol..

[38] Lombardi T., Bernardello F., Berton F., Porrelli D., Rapani A., Camurri Piloni A., Fiorillo L., Di Lenarda R., Stacchi C. (2018). Efficacy of Alveolar Ridge Preservation after Maxillary Molar Extraction in Reducing Crestal Bone Resorption and Sinus Pneumatization: A Multicenter Prospective Case-Control Study. Biomed. Res. Int..

[39] Fiorillo L., De Stefano R., Cervino G., Crimi S., Bianchi A., Campagna P., Herford A.S., Laino L., Cicciù M. (2019). Oral and Psychological Alterations in Haemophiliac Patients. Biomedicines.

[40] Sambataro S., Cervino G., Fiorillo L., Cicciu M. (2018). Upper First Premolar Positioning Evaluation for the Stability of the Dental Occlusion: Anatomical Considerations. J. Craniofac. Surg..

[41] Wroe A.L., Bowers H.M. (2019). Beliefs about sharing illness experiences: Development of a scale and relationship with symptoms of fibromyalgia. Br. J. Health Psychol..

[42] Tesio V., Ghiggia A., Di Tella M., Castelli L. (2019). Utility of the Diagnostic Criteria for Psychosomatic Research in assessing psychological disorders in fibromyalgia patients. J. Affect. Disord..

[43] Onder H., Hamamci M., Alpua M., Ulusoy E.K. (2019). Comorbid fibromyalgia in migraine patients: Clinical significance and impact on daily life. Neurol. Res..

[44] Minerbi A., Gonzalez E., Brereton N.J.B., Anjarkouchian A., Dewar K., Fitzcharles M.A., Chevalier S., Shir Y. (2019). Altered microbiome composition in individuals with fibromyalgia. Pain.

[45] Jacobs H., Bockaert M., Bonte J., D’Haese M., Degrande J., Descamps L., Detaeye U., Goethals W., Janssens J., Matthys K. (2019). The Impact of a Group-Based Multidisciplinary Rehabilitation Program on the Quality of Life in Patients With Fibromyalgia: Results From the QUALIFIBRO Study. J. Clin. Rheumatol..

[46] Stacchi C., Lombardi T., Cusimano P., Berton F., Lauritano F., Cervino G., Di Lenarda R., Cicciù M. (2017). Bone Scrapers Versus Piezoelectric Surgery in the Lateral Antrostomy for Sinus Floor Elevation. J. Craniofac. Surg..

[47] Cicciù M., Herford A.S., Cervino G., Troiano G., Lauritano F., Laino L. (2017). Tissue fluorescence imaging (VELscope) for quick non-invasive diagnosis in oral pathology. J. Craniofac. Surg..

[48] Isola G., Cicciu M., Fiorillo L., Matarese G. (2017). Association Between Odontoma and Impacted Teeth. J. Craniofac. Surg..

[49] Lo Giudice G., Cutroneo G., Centofanti A., Artemisia A., Bramanti E., Militi A., Rizzo G., Favaloro A., Irrera A., Lo Giudice R. (2015). Dentin morphology of root canal surface: A quantitative evaluation based on a scanning electronic microscopy study. BioMed Res. Int..

[50] Herford A.S., Lu M., Akin L., Cicciù M. (2012). Evaluation of a porcine matrix with and without platelet-derived growth factor for bone graft coverage in pigs. Int. J. Oral Maxillofac. Implants.

[51] Maiorana C., Beretta M., Grossi G.B., Santoro F., Herford A.S., Nagursky H., Cicciù M. (2011). Histomorphometric evaluation of anorganic bovine bone coverage to reduce autogenous grafts resorption: Preliminary results. Open Dent. J..

[52] Cicciù M., Herford A.S., Stoffella E., Cervino G., Cicciù D. (2012). Protein-signaled guided bone regeneration using titanium mesh and Rh-BMP2 in oral surgery: A case report involving left mandibular reconstruction after tumor resection. Open Dent. J..

[53] Giudice G., Cicciù M., Cervino G., Lizio A., Visco A. (2012). Flowable resin and marginal gap on tooth third medial cavity involving enamel and radicular cementum: A SEM evaluation of two restoration techniques. Indian J. Dent. Res..

[54] Cervino G., Fiorillo L., Herford A.S., Romeo U., Bianchi A., Crimi S., D’Amico C., De Stefano R., Troiano G., Santoro R. (2019). Molecular Biomarkers Related to Oral Carcinoma: Clinical Trial Outcome Evaluation in a Literature Review. Dis. Markers.

[55] Cervino G., Fiorillo L., Laino L., Herford A.S., Lauritano F., Giudice G.L., Fama F., Santoro R., Troiano G., Iannello G. (2018). Oral Health Impact Profile in Celiac Patients: Analysis of Recent Findings in a Literature Review. Gastroenterol. Res. Pract..

[56] Giudice G., Lipari F., Lizio A., Cervino G., Cicciù M. (2012). Tooth fragment reattachment technique on a pluri traumatized tooth. J. Conserv. Dent..

[57] Crimi S., Fiorillo L., Bianchi A., D’Amico C., Amoroso G., Gorassini F., Mastroieni R., Marino S., Scoglio C., Catalano F. (2019). Herpes Virus, Oral Clinical Signs and QoL: Systematic Review of Recent Data. Viruses.

[58] Cervino G., Terranova A., Briguglio F., De Stefano R., Famà F., D’Amico C., Amoroso G., Marino S., Gorassini F., Mastroieni R. (2019). Diabetes: Oral health related quality of life and oral alterations. Biomed. Res. Int..

[59] Troiano G., Laino L., Cicciu M., Cervino G., Fiorillo L., D’Amico C., Zhurakivska K., Lo Muzio L. (2018). Comparison of Two Routes of Administration of Dexamethasone to Reduce the Postoperative Sequelae After Third Molar Surgery: A Systematic Review and Meta-Analysis. Open Dent. J..

[60] Laino L., Cicciù M., Fiorillo L., Crimi S., Bianchi A., Amoroso G., Monte I.P., Herford A.S., Cervino G. (2019). Surgical Risk on Patients with Coagulopathies: Guidelines on Hemophiliac Patients for Oro-Maxillofacial Surgery. Int. J. Environ. Res. Public Health.

[61] Fiorillo L. (2019). Chlorhexidine Gel Use in the Oral District: A Systematic Review. Gels.

[62] Stefano R.D., Bruno A., Muscatello M., Cedro C., Cervino G., Fiorillo L. (2019). Fear and anxiety managing methods during dental treatments: Systematic review of recent data. Minerva Stomatol..

[63] De Stefano R. (2019). Psychological Factors in Dental Patient Care: Odontophobia. Medicina.

[64] Zoccali R., Muscatello M.R., Bruno A., Barilla G., Campolo D., Meduri M., Familiari L., Bonica M., Consolo P., Scaffidi M. (2006). Anger and ego-defence mechanisms in non-psychiatric patients with irritable bowel syndrome. Dig. Liver Dis..

[65] Muscatello M.R., Bruno A., Scimeca G., Pandolfo G., Zoccali R.A. (2014). Role of negative affects in pathophysiology and clinical expression of irritable bowel syndrome. World J. Gastroenterol..

[66] Muscatello M.R., Bruno A., Pandolfo G., Mico U., Stilo S., Scaffidi M., Consolo P., Tortora A., Pallio S., Giacobbe G. (2010). Depression, anxiety and anger in subtypes of irritable bowel syndrome patients. J. Clin. Psychol. Med. Settings.

[67] Muscatello M.R., Bruno A., Mento C., Pandolfo G., Zoccali R.A. (2016). Personality traits and emotional patterns in irritable bowel syndrome. World J. Gastroenterol..

